# Motor planning error in Parkinson's disease and its clinical correlates

**DOI:** 10.1371/journal.pone.0202228

**Published:** 2018-08-13

**Authors:** Tsubasa Kawasaki, Kyohei Mikami, Tsutomu Kamo, Ryoma Aoki, Rumiko Ishiguro, Hiroshi Nakamura, Ryosuke Tozawa, Nao Asada, Yukinobu Hiiragi, Yoichi Yamada, Masahiro Hirano, Kazuko Katsuki

**Affiliations:** Institute of Sports Medicine and Science, Tokyo International University, Matoba, Kawagoe-shi, Saitama, Japan; Chinese University of Hong Kong, HONG KONG

## Abstract

This study aimed to investigate (a) motor planning difficulty by using a two-step test in Parkinson’s disease (PD) compared with age-matched healthy subjects and (b) the relationship between motor planning difficulty and clinical factors. The two-step test was performed by 58 patients with PD with Hoehn & Yahr (H&Y) stage I–IV and 110 age-matched healthy older adult controls. In the two-step test, the participants estimated the two-step distance with maximum effort. Subsequently, they performed the actual two-step trial to measure the actual maximum distance. We calculated the accuracy of the estimation (estimated distance minus actual distance). In both groups, subjects who estimated >5 cm were defined as the overestimation group, and those who estimated <5 cm over- and underestimation were defined as the non-overestimation group. The overestimation group consisted of 17 healthy older adults (15.5%) and 23 patients with PD (39.7%). The number of patients with PD with overestimation was significantly more than that of healthy controls by Chi-squared test. H&Y stage and the Unified Parkinson’s Disease Rating Scale (UPDRS) part II and III scores in overestimation group in PD patients were significantly higher than those in overestimation group in PD patients. Moreover, multiple regression using H&Y stage and UPDRS parts II and III as independent variables showed that the UPDRS part II score was the only related factor for the estimation error distance. Estimation error distance was significant correlated with UPDRS parts II and III. Patients with PD easily have higher rates of motor-related overestimation than age-matched healthy controls. In addition, UPDRS parts II and III expressed ability of activities of daily living and motor function as influences on motor-related overestimation. Particularly, multiple regression indicated that UPDRS part II directly showed the ability of daily living as an essential factor for overestimation.

## Introduction

Patients with Parkinson’s disease (PD) present with motor disorders, such as resting tremor, bradykinesia, rigidity, and postural instability [1], and other typical motor symptoms showing abnormal gait freezing gait, and motor coordination deficits [1, 2]. Some cognitive dysfunctions may be observed in addition to these motor symptoms. A representative cognitive dysfunction is decline in executive function, which is a part of frontal brain function and refers to the cognitive control of behavior: selecting and successfully monitoring behaviors that facilitate the attainment of chosen goals. Lezak has stated that executive function includes goal setting, motor planning, and effective motor execution [3]. Some previous studies reported that individuals with PD have decreased brain ability in the frontal lobe and striatum [4, 5], thus influencing their cognitive dysfunction [6–8].

In the present study, we focused on the executive function of motor prediction based on working memory and motor-related strategic thinking. In a previous study, although movement accuracy did not decline, they prolonged the time until movement by using the Tower of London task [9]. Based on the results, patients with PD have been suggested to have difficulty in the motor prediction of operating figures [9]. Furthermore, Owen et al. then focused on severe PD in the investigation of decreased motor prediction and reported that patients with severe PD particularly had a difficulty in motor execution accuracy of the task in addition to motor prediction delay [10]. These results suggested that severity of PD affected motor prediction and function.

The effects of motor prediction disorder in patients with PD are shown not only in operation of figures but also in action imitation tasks. For example, using apraxia testing, Goldenberg et al. have reported that patients with PD had impaired ability of action imitation [11]. These previous studies have shown that patients with PD had difficulty in motor prediction by using executive function.

Difficulty of motor prediction affect some actual movement disabilities. Cohen et al. reported that patients with PD frequently made a judgment error on whether they could pass an aperture without collisions. This result showed the difficulty of motor prediction in patients with PD, indicating that the judgment error would result in freezing gait [12]. Kameda et al. measured the amounts of discordance between motor prediction and motor execution of forward reach distance (functional reach distance), reporting that overestimation (prediction distance > actual distance) leads to a high frequency of falls in patients with PD [13]. Through these studies, movement disabilities in patients with PD have been suggested to be related to motor prediction error, including motor-related overestimation.

However, whether a factor is associated with motor prediction error is still unclear. Identification of the factor associated with motor prediction error would contribute in the determination of the mechanisms of movement disability (freezing gait or falling) directly affecting activity of daily living. In the present study, we used a two-step test to measure motor prediction error [14]. The reliability of the two-step test, which was developed to judge the requirement of nursing care in Japan, or being at risk of doing so, due to a decline in mobility, resulting from a musculoskeletal system disorder, has been confirmed [14]. Subsequently, we investigated whether motor prediction was related to the factors of PD (severity, duration, cognitive function, or motor function).

## Materials and methods

### Subjects

Overall, 58 patients with PD (26 men and 32 women; mean age, 73.0 ± 7.4 years) and 110 age-matched healthy elderly subjects (21 men and 89 women; mean age, 72.8 ± 6.7 years) participated in the present study. Cognitive function was assessed using the Mini-Mental State Examination (MMSE) and Six-item test. MMSE was used for patients with PD and elderly subjects, and six-item score was used only for elderly subjects. Note that, for control subjects, the six-item score was used as a screening for dementia. Subjects who scored fewer than three points in the six-item test were administered the MMSE to assess details of their cognitive function. In all patients with PD, MMSE was first used to accurately assess cognitive function following traditional methods. The exclusion criteria were (a) cognitive impairment MMSE score <23 [15], and six-item score <3 [16]); and (b) visual impairment influence with action. In addition to the criteria, Hoehn and Yahr (H&Y) stage >4 was excluded for patients with PD [17]. These criteria were set to carry out a motor-prediction task exactly using a two-step-forward task (see below for details). The tenets of the Declaration of Helsinki were followed, and participants provided informed consent before study participation.

### Procedure

**Fig 1:**
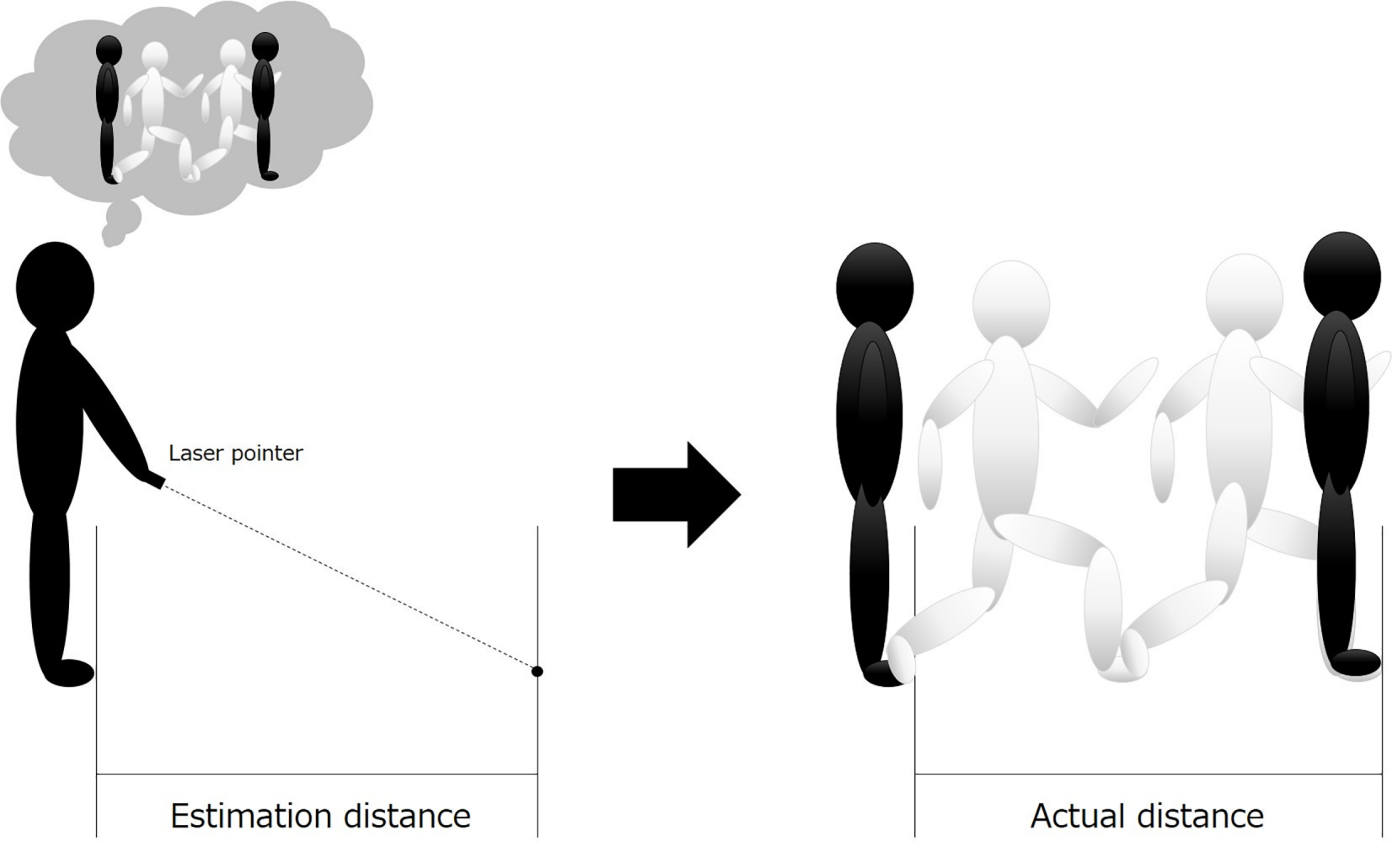
Schematic illustration of the two-step test. Participants estimated the two-step distance forward with maximum effort and indicated the distance using a laser pointer. After the estimation, the actual distance was measured.

First, the subjects performed a two-step test (measuring estimation and actual distance) (Fig 1). All subjects were instructed to predict the distance that they can perform two-step forward as far as possible with maximum effort. The subjects indicated the prediction point using a laser pointer, and the examiner measured the predicted distance. The actual two-step distance was subsequently measured. When the subjects performed the actual two-step performance, they were instructed to put their legs together to stand still before and after taking the two steps forward. For each patient, we also identified Unified Parkinson’s Disease Rating Scale (UPDRS) part I–III scores [18], H&Y stage, MMSE score, and L-dopa dose (mg/day).

### Statistical analyses

The estimation error (estimation distance [cm] minus actual distance [cm]) was calculated. The subjects with accuracy of estimation of >5 cm and <5 cm were classified into the overestimation and non-overestimation groups, respectively. The criteria of this definition were derived from evidence that the prediction distance using the laser pointer had a measurement margin of error of 5 cm in our unpublished preliminary study. Exactly indicating the estimation distance was difficult because of tremors in some patients with PD. That is, we believe that some margin (±5 cm) is necessary in the judgment of overestimation or non-overestimation.

For preliminary analyses, to demonstrate whether gender influences overestimation, the number of female subjects with overestimation was analyzed using Pearson’s Chi-squared test (*X*^2^) with Yates’ correction. This is why the gender ratio was completely different in the two groups (patients with PD and healthy control groups); there were more females in the control group than in the PD group.

Pearson’s Chi-squared test (*X*^2^) with Yates’ correction was also conducted to examine whether patients with PD had overestimation as compared with healthy control subjects. The number of subjects with overestimation was analyzed in the two groups of subjects (patients with PD vs. healthy control subjects).

A one-way analysis of variance (ANOVA) followed by the Bonferroni post hoc test was conducted with groups (overestimated vs. non-overestimated PD group vs. overestimated control group vs. non-overestimated control group) regarding estimation distance, actual distance, and estimation error distance to determine details of the overestimation in patients with PD.

For each UPDRS (I–III) score, the Mann–Whitney U test was performed to analyze the H&Y stage, duration of PD, L-dopa dose, and MMSE. Variables showing significant difference in the statistical analyses were used for Pearson’s correlation analysis with estimation error and also utilized as predictor variables in multiple regression of estimation error distance.

We used SPSS for Windows (version 17.0; SPSS Inc., Chicago. IL, USA) for all statistical analyses and set the statistical significance at p < 0.05. This study was approved by the Ethics Committee of the Japan Primary Care Association (approval, 2017–006) and Ryotokuji University (approval, 2829).

## Results

### Preliminary results and one implication

Rate of female and male in overestimation group in PD patients was 60.9% (14/23) and 39.1% (9/23) respectively. No significant gender difference was observed by the Pearson’s Chi-squared (p = 0.12). In the same manner as PD patients, the rate of female and male in overestimation group in control subjects was 70.6% (12/17) and 29.4% (5/17), respectively. No significant gender differences were observed by the Pearson’s Chi-squared (p = 0.24). This suggests that there was no possibility for impact of gender on overestimation.

### Main results

The characteristics of all subjects and results of the two-step test are shown in Table 1. The rates of overestimation in PD patients and control subjects were 39.7% (23/58) and 15.5% (17/110), respectively. The Pearson’s Chi-squared test showed that the percentage of overestimation in patients with PD was significantly higher than that in age-matched healthy control subjects (p = 0.005).

**Table 1:** Demographic clinical details and results of two-step test of control groups (non-overestimation and overestimation) and patients with PD groups (non-overestimation and overestimation) (mean ± *SD*).

	Controls		PD patients	
	Overestimation	Non-overestimation	Overestimation	Non-overestimation
Number	17	93	23	35
Age (years)	72.33 ± 6.28	75.35 ± 8.57	73.5 ± 8.26	71.72± 8.98
Gender (female)	12	77	14	14
MMSE	― ^§^	― ^§^	27.57 ± 2.37	28.11 ± 1.95
Hoehn-Yahr stage	―	―	2.91± 0.70 ^‡^	2.50± 0.70
Duration of disease(months)	―	―	7.87 ± 6.02	5.77 ± 5.75
Drugs (levodopa equivalent doses (mg)	―	―	443.18 ± 164.24	366.18 ± 154.10
UPDRS partⅠ	―	―	2.87 ± 2.16	2.03 ± 1.76
UPDRS part Ⅱ	―	―	10.87± 5.35 ^‡^	6.46 ± 3.97
UPDRS part Ⅲ	―	―	23.65± 10.71 ^‡^	15.20 ± 9.90
Estimation distance (cm)	237.94 ± 33.21	179.84 ± 31.90 *	163.26 ± 41.69 *	151.86 ± 38.39 *^,^ ^†^
Actual distance (cm)	210.53 ± 24.94	210.68 ± 30.44	139.35 ± 41.04 *^,^ ^†^^,^ ^‡^	175.14 ± 40.28 *^,^ ^†^
Estimation error distance (cm)	27.41± 18.22	-30.84 ± 22.90 *	23.91 ± 16.54 ^†^^,^ ^‡^	-20.29 ± 18.19 *^,^ ^†^

* comparing with overestimation group in control subjects (p < .05)

^†^ comparing with non-overestimation group in control subjects (p < .05)

^‡^ comparing with non-overestimation group in PD patients (p < .05): Bonferroni correction was applied.

^§^ Six-item score was used instead of MMSE to screen for dementia.

MMSE: Mini Mental State Examination; UPDRS: Unified Parkinson’s Disease Rating Scale

With regard to estimation distance, a one-way ANOVA showed a significant main effect of the group F(3, 167) = 24.72, p < 0.0001. Post-hoc analysis showed that estimation distance in the overestimation and non-overestimation groups in patients with PD and non-overestimation group in control subjects was lesser than the overestimation group in control subjects (overestimation group in patients with PD vs. that in control subjects: t(164) = 6.69, p < 0.0001; non-overestimation group in PD patients vs. overestimation group in control subjects: t(164) = 8.36, p < 0.0001); non-overestimation group vs. overestimation group in control subjects: t(164) = 6.31, p < 0.0001). Non-overestimation group in control subjects was significant greater than that in PD patients: t(164) = 4.04, p < 0.0001), whereas no significant differences between the overestimation and non-overestimation groups in PD patients, and between overestimation group in PD patients and non-overestimation group in control subjects were observed: t(165) = 1.22, p = 0.23; t(164) = 2.04, p = 0.04, respectively (Table 1).

With regard to estimation distance, a one-way ANOVA showed a significant main effect of the group F(3, 167) = 24.72, p < 0.0001. Post hoc analysis showed that estimation distances in the overestimation and non-overestimation groups in patients with PD and the non-overestimation group in control subjects were less than that in the overestimation group in control subjects (overestimation group in patients with PD vs. that in control subjects: t(164) = 6.69, p < 0.0001; non-overestimation group in PD patients vs. overestimation group in control subjects: t(164) = 8.36, p < 0.0001); non-overestimation group vs. overestimation group in control subjects: t(164) = 6.31, p < 0.0001). The estimation distance in the non-overestimation group in control subjects was significantly greater than that in PD patients: t(164) = 4.04, p < 0.0001), whereas there were no significant differences between the overestimation and non-overestimation groups in PD patients or between the overestimation group in PD patients and the non-overestimation group in control subjects: t(165) = 1.22, p = 0.23; t(164) = 2.04, p = 0.04, respectively (Table 1).

With regard to actual distance, a one-way ANOVA showed a significant main effect of the group F(3, 167) = 31.99, p < 0.0001. Post hoc analysis showed that actual distance in the overestimation and non-overestimation groups of PD patients was significantly less than that in the overestimation group in control subjects (t(164) = 6.53, p < 0.0001; t(164) = 3.51, p = 0.0006, respectively). The actual distance in the overestimation group in PD patients was significantly smaller than those in the non-overestimation group in PD patients and control subjects (t(164) = 3.91, p < 0.0001; t(164) = 8.98, p < 0.0001, respectively). The actual distance in the non-overestimation group in PD patients was significantly smaller than that in control subjects (t(164) = 5.25, p < 0.0001) (Table 1). There was no significant difference between overestimation and non-overestimation groups in control subjects (t(164) = 0.02, p = 0.99).

With regard to the estimation error distance, a one-way ANOVA also showed a significant main effect of the group F(3, 167) = 69.23, p < 0.0001. Post hoc analysis showed no significant difference between the overestimation groups in PD patients and control subjects (t(164) = 0.53, p = 0.60)) and between non-overestimation groups in PD patients and control subjects (t(164) = 2.56, p = 0.01). On the other hand, the estimation error distances in the overestimation groups of PD patients and control subjects were significantly larger than that in the non-overestimation group in control subjects (t(164) = 11.31, p < 0.0001; t(164) = 10.63, p < 0.0001, respectively). Similarly, the estimation error differences in the overestimation groups in PD patients and control subjects were significantly larger than that in the non-overestimation group in PD patients (t(164) = 7.92, p < 0.0001; t(164) = 7.76, p < 0.0001, respectively).

The identified data were compared between the overestimation and non-overestimation groups in patients with PD. Table 1 also shows the result of comparisons between the overestimation and non-overestimation groups. The result showed that the H&Y stage (p = 0.04) and UPDRS parts II (p = 0.002) and III (p = 0.005) in the overestimation group were significantly higher than those in the non-overestimation group. In contrast, no difference was found between the two groups in MMSE (p = 0.34), duration of PD (p = 0.19), L-dopa dose (p = 0.08), and UPDRS part I (p = 0.15).

**Fig 2:**
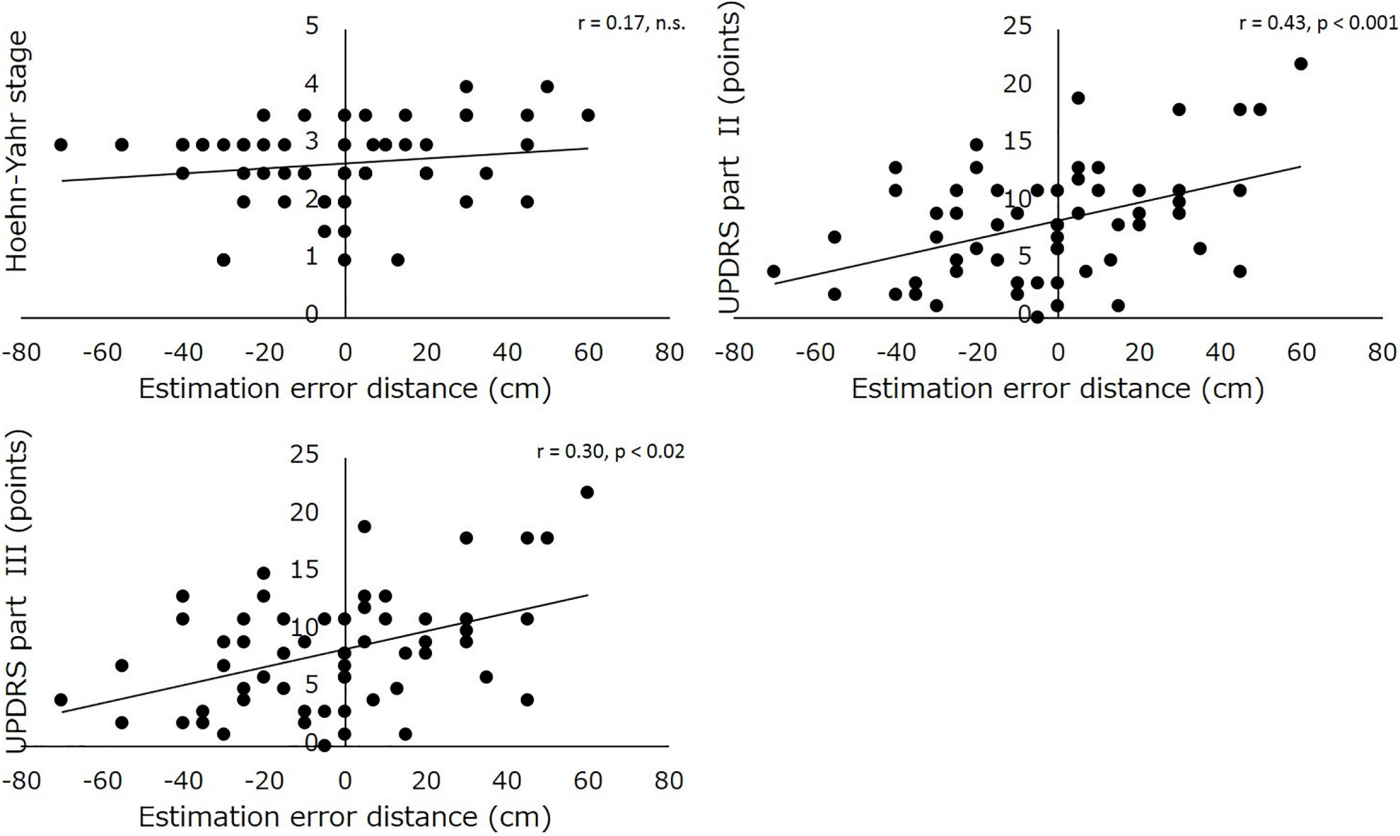
Scattergrams of the estimation error distance, and H&Y stage (upper left), and UPDRS part II (upper right) and III (lower).

The H&Y stage and UPDRS parts II and III showed significant differences between the two groups in patients with PD, and these were used for Pearson’s correlation analysis with estimation error. Results showed that the estimation error significantly correlated with UPDRS part II and III scores (r = 0.43 and 0.30, respectively), but not at the H&Y stage (r = 0.17) (Fig 2).

Also, the three dependent variables were also used as independent variables in a linear multiple regression analysis. Results showed the regression equation obtained in the estimation error distance (−22.689 + 2.428 × UPDRS part II score, R^2^ = 0.19). The UPDRS part II score was significantly associated with the estimation error (p < 0.001).

## Discussion

In the present study, we verified motor prediction error in patients with PD and the association between the motor prediction error and clinical factors. The result showed that more patients with PD overestimated their own motor performance compared with healthy control subjects. This result was supported by a previous report that showed the decreased ability of motor prediction in patients with PD: a previous study verifying motor prediction error in patients with PD using forward reach task (functional reach task) reported that they estimated higher motor performance than actual motor performance comparing with age-matched healthy control subjects [13]. The results in the previous report were consistent with the present study. This confirms that the two-step test could be available to measure the accuracy of motor estimation, and that patients with PD have overestimation compared with healthy control subjects.

Results in accuracy of estimated distance compared among four groups (overestimation and non-overestimation × PD patients and control subjects) showed that the estimation error distances of overestimated PD patients and control subjects were higher than those of the other two groups (non-overestimation groups in PD patients and control subjects). The estimation distance was not significantly different among the two PD groups and the non-overestimation group in control subjects, whereas, the actual distance of the overestimation group in PD patients was significantly smaller than that of non-overestimation groups in PD patients and control subjects, i.e., the two-step performance of the overestimation group in PD patients was significantly lower than that in the non-overestimation group. Based on the results, overestimation in PD patients is suggested to be caused by the decrease of motor performance.

Consistent with previous studies [19, 20], overestimation was observed in healthy elderly people; as stated above, the number of PD patients who overestimated was significantly more than that in healthy controls. Taken together with these results, this suggests that overestimation in PD subjects would be caused by the onset of PD in addition to the effects of natural aging.

Comparing the overestimation group with the non-overestimation group in identified data, significant higher H&Y stage and UPDRS parts II and III were shown, whereas duration of disease, L-dopa dose, MMSE, and UPDRS part I were not different. These results suggested that the accuracy of motor estimation was involved in the H&Y stage and UPDRS parts II and III. These variables expressed the severity of PD associated with motor function. Variables that showed no significant difference (i.e., L-dopa dose, MMSE, and UPDRS part I) were consistently non-motor function-related. H&Y stage showed the severity of PD as assessed by motor symptoms. UPDRS parts II and III expressed the ability of activity of daily living and movement disorder, respectively. Considering characteristics of these variables, the accuracy of motor estimation is associated with severity of PD involved in motor function. Although these statements are plausible, the lack of correlation between the estimation error distance and H&Y stage by Pearson’s correlation analysis must be considered; this distance was correlated with only UPDRS parts II and III. This supports an association between estimation error and ability of activity of daily living and movement disorder, whereas concluding that the degree of estimation error correlates with the severity of PD would be premature. Future research is needed to additionally investigate the relationship between estimation error and the H&Y stage, i.e., the severity of PD.

The reason for the association between the accuracy of motor estimation and progress of motor-related symptoms may involve difficulty of upload (recognition). This decreases their motor performance based on the progress of PD. Generally, progress of PD leads to low physical activity, which means that patients with PD are considered to lose their ability of uploading their motor performance. Sakurai et al. has reported that older adults with high risk of falling have overestimation in the step-over test, suggesting that poor physical activity might be avoided through participation in physical activity in daily living, and these lifestyles would limit the recognition of the current activity [20]. Taking these statements together, patients with PD tend to maintain their own recognition of current motor performance (i.e., current motor performance was not uploaded before onset of PD). As a result, patients with severe PD have low motor performance and overestimation.

Particularly, multiple regression analysis showed that the accuracy of motor estimation significantly was associated with UPDRS part II. The coefficient of multiple determination (R^2^) value was 0.19, which was relatively low; however, the standardized regression coefficient (*β*) value was 0.43, suggesting that the accuracy of motor estimation and UPDRS part II expressed that the ability of daily living has a medial relationship [21]. A previous study showed that the executive function in PD was related to UPDRS part II [8]. The present study was consistent with the previous study. This showed that executive function, including motor estimation, is involved in UPDRS part II, which expresses the ability of activity of daily living that is required to sequence motion based on motor planning (executive function). In the two-step test procedure, the patients with PD were also instructed to execute after estimating their own performance. Therefore, UPDRS part II would be shown as a predictor variable because this procedure included the process of motor planning (executive function).

In conclusion, the present study determined that (a) the frequency of overestimation in patients with PD was higher than that in healthy elderly people, and (b) the overestimation in PD was associated with motor-related severely (H&Y stage and UPDRS parts II and III). These results suggest that overestimation (which often causes falls) may be influenced by the progress of motor-related symptoms in patients with PD. However, whether interventions that address motor-related symptoms directly contribute to fall prevention remains unclear. Future studies should be conducted on the prevention effects of motor-related symptom interventions, especially with respect to falling risk in patients with PD.
