# Correction: Motor planning error in Parkinson’s disease and its clinical correlates

**DOI:** 10.1371/journal.pone.0228698

**Published:** 2020-01-30

**Authors:** Tsubasa Kawasaki, Kyohei Mikami, Tsutomu Kamo, Ryoma Aoki, Rumiko Ishiguro, Hiroshi Nakamura, Ryosuke Tozawa, Nao Asada, Yukinobu Hiiragi, Yoichi Yamada, Masahiro Hirano, Kazuko Katsuki

Fig 2 is incorrect. The authors have provided a corrected version here.

**Fig 2:**
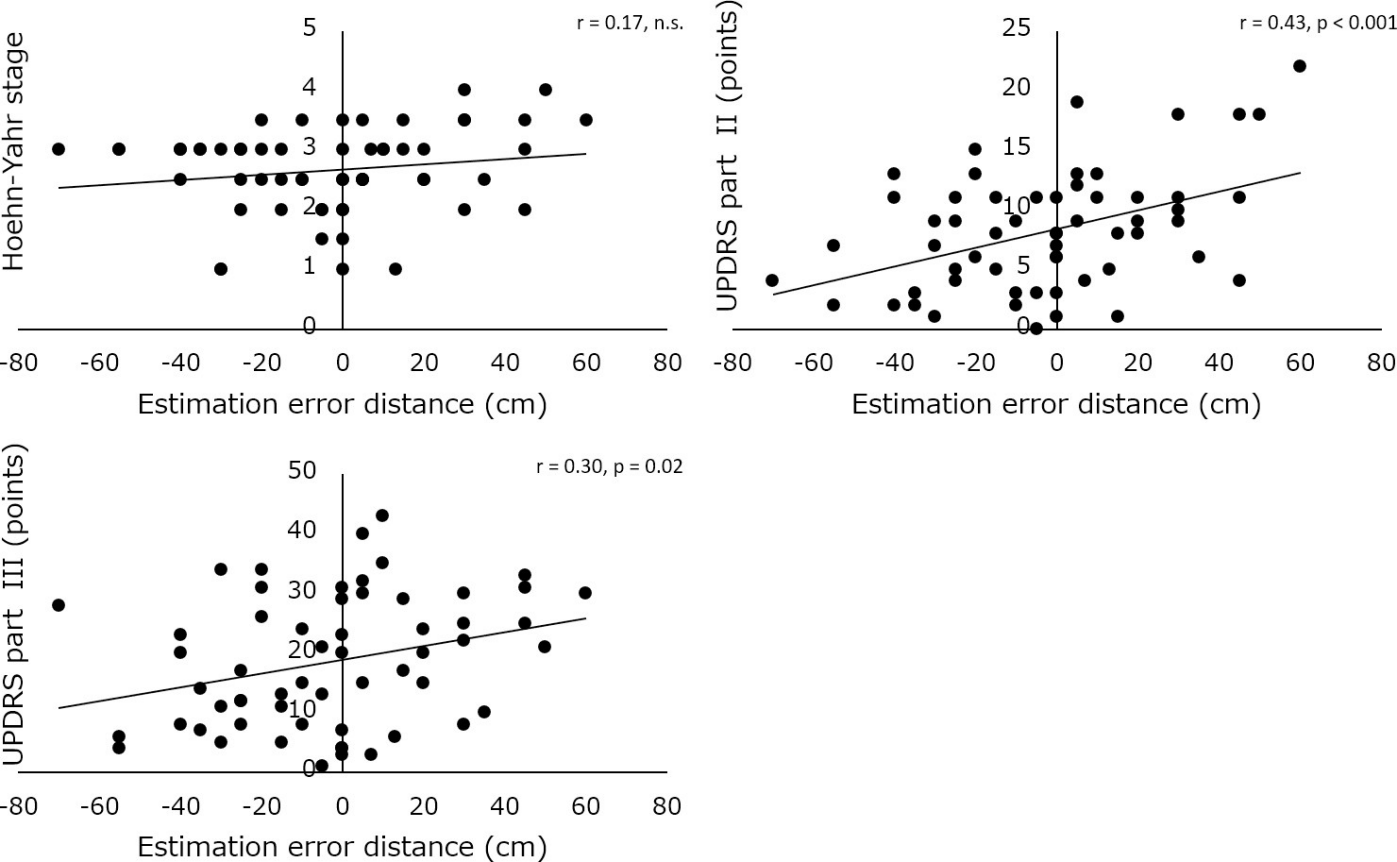
Scattergrams of the estimation error distance, and H&Y stage (upper left), and UPDRS part II (upper right) and III (lower).
